# An enrichment protocol and analysis pipeline for long read sequencing of the hepatitis B virus transcriptome

**DOI:** 10.1099/jgv.0.001856

**Published:** 2023-05-17

**Authors:** Esther Ng, Mihaela-Olivia Dobrica, James M. Harris, Yanxia Wu, Senko Tsukuda, Peter A. C. Wing, Paolo Piazza, Peter Balfe, Philippa C. Matthews, M. Azim Ansari, Jane A. McKeating

**Affiliations:** ^1^​ Nuffield Department of Medicine, University of Oxford, Oxford, UK; ^2^​ Wellcome Centre for Human Genetics, University of Oxford, Oxford, UK; ^3^​ Chinese Academy of Medical Sciences Oxford Institute, University of Oxford, Oxford, UK; ^4^​ The Francis Crick Institute, London, UK; ^5^​ Division of Infection and Immunity, University College London, London, UK; ^6^​ Peter Medawar Building for Pathogen Research, Nuffield Department of Medicine, University of Oxford, Oxford, UK; ^§^​ Present address: Institute of Biochemistry of the Romanian Academy, Bucharest, Romania

**Keywords:** HBV, long read sequencing, PacBio, transcriptome assembly, RNA splicing

## Abstract

Hepatitis B virus (HBV) is one of the smallest human DNA viruses and its 3.2 Kb genome encodes multiple overlapping open reading frames, making its viral transcriptome challenging to dissect. Previous studies have combined quantitative PCR and Next Generation Sequencing to identify viral transcripts and splice junctions, however the fragmentation and selective amplification used in short read sequencing precludes the resolution of full length RNAs. Our study coupled an oligonucleotide enrichment protocol with state-of-the-art long read sequencing (PacBio) to identify the repertoire of HBV RNAs. This methodology provides sequencing libraries where up to 25 % of reads are of viral origin and enable the identification of canonical (unspliced), non-canonical (spliced) and chimeric viral-human transcripts. Sequencing RNA isolated from *de novo* HBV infected cells or those transfected with 1.3 × overlength HBV genomes allowed us to assess the viral transcriptome and to annotate 5′ truncations and polyadenylation profiles. The two HBV model systems showed an excellent agreement in the pattern of major viral RNAs, however differences were noted in the abundance of spliced transcripts. Viral-host chimeric transcripts were identified and more commonly found in the transfected cells. Enrichment capture and PacBio sequencing allows the assignment of canonical and non-canonical HBV RNAs using an open-source analysis pipeline that enables the accurate mapping of the HBV transcriptome.

## Introduction

An estimated two billion people globally have serological evidence of exposure to hepatitis B virus (HBV) with an estimated 275 million diagnosed infections [1]. Chronic hepatitis B can lead to progressive liver disease, cirrhosis, and hepatocellular carcinoma. HBV has a small circular 3.2 Kb DNA genome that is classified into eight established genotypes (A-H) on the basis of sequence diversity, together with two further putative genotypes, I and J [2, 3]. Differences in viral replication and natural history across HBV genotypes have been reported, however the implications for disease progression or treatment are not well understood [4, 5].

The HBV genome relies on a finely coordinated pattern of transcription from its four overlapping open reading frames [6, 7]. The viral covalently closed circular DNA (cccDNA) encodes four promoters (basal core promoter, Sp1, Sp2 and Xp) that are transcribed by the host RNA polymerase II complex to generate six major viral RNAs with heterogeneous 5′ ends but a common 3′ polyadenylation signal (PAS) [8]. These overlapping RNAs include: pre-core (preC, 3.5 Kb) that encodes e antigen; a pre-genome (pgRNA, 3.5 Kb), which is translated to yield the core and polymerase proteins; the preS1, preS2 and S surface protein encoding RNAs (2.4, 2.1 and 2.0 Kb respectively), and the transcript for the multi-functional X protein (HBx, 0.7 Kb). Several spliced HBV RNAs derived from pgRNA or preS2/S have been reported [9, 10] and have been associated with the development of hepatocellular carcinoma [10, 11], however, their role in the viral life cycle is not well understood. The pgRNA is reverse transcribed by the viral polymerase to generate new partially circularised DNA genomes that can be secreted as infectious particles [12]. Aberrant reverse transcription can generate a double stranded linear DNA (dslDNA) that can integrate into the host genome [13]. This integration process separates the basal core promoter from its downstream transcription start sites (TSS) and prevents the genesis of preC or pgRNAs. Although these integrations are a replicative ‘dead end’ for the virus, they can transcribe S antigens and HBx and have been reported to play a role in viral oncogenesis [14, 15].

The earliest method to identify HBV transcripts was physical visualisation by northern blot, which identified the lengths of the major RNAs [16]. However, this approach requires large amounts of starting material and does not lend itself to accurate quantification. Recent quantitative approaches include RT-qPCR [17], digital droplet PCR [18] and 5′-RACE [8] that target specific regions of the genome. However, care is needed to identify the overlapping HBV transcripts as variation in PCR amplification efficiency can bias the inferred RNA levels. PCR approaches to quantify only preC/pg RNAs and total viral RNAs are likely to underestimate the complexity of the viral transcriptome and overlook the presence of spliced RNAs. Next generation RNA sequencing methods such as Illumina are based on fragmenting the template into short (150–300 bp) pieces which provides excellent sequence accuracy, but precludes the assignment of reads to the original viral transcripts. As the quantity of HBV RNAs in clinical samples is often low, in the range of 200 viral transcripts/million reads, large numbers of sequences (over 100 million reads) are required to obtain robust data sets [19]. Long read sequencing approaches (Oxford Nanopore and PacBio) can identify full-length transcripts, however, the low number of total sequences obtained, combined with the limited amounts of viral RNA in the starting material can limit their application. To date, long read methods have been used to sequence HBV DNA in plasma or serum samples containing high viral loads (>10^8^ HBV DNA copies ml^−1^) [20–23]. Recent studies to sequence rare host mRNAs have enriched transcripts in cDNA mixtures using biotinylated oligonucleotide probe-based capture [24–26]. van Buuren and colleagues applied an enrichment protocol to identify chimeric HBV-human transcripts in CHB liver biopsies and used long read sequencing to identify clonally expanded HBV-associated chromosomal rearrangements [27].

We used HBV specific oligonucleotide probes to enrich viral cDNA for PacBio Sequel II sequencing and developed in-house analytical pipelines for mapping the viral transcriptome. We compared the HBV transcriptome from infected or transfected human hepatoma cells (HepG2-NTCP). Lipid-based delivery of plasmids encoding overlength HBV genomes have been used for decades to study HBV transcription, where RNAs can be derived from either plasmid or cccDNA templates [28]. Transfecting HBV plasmids or minigenomes directly into cells provides a simple method to generate high quantities of infectious HBV, however, the impact of delivering a large bolus of viral DNA into cells on the resulting viral transcriptome is not known. In this study we exploited an enrichment capture method combined with PacBio long-read sequencing to perform in depth mapping of the HBV transcriptome and show comparable transcript abundance in both HBV infected and transfected cells.

## Methods

### Transfection and HBV infection of HepG2-NTCP cells

HepG2-NTCP cells (RRID:CVCL_JY40) were maintained in Dulbecco’s modified Eagle’s medium (DMEM) supplemented with 10 % fetal bovine serum (FBS), 2 mM l-glutamine, 1 mM sodium pyruvate, 50 U ml^−1^ penicillin/ streptomycin, and non-essential amino acids (Thermo Fisher Scientific, Paisley, UK) and maintained in a 5 % CO_2_ atmosphere at 37 °C. Cells at 80 % confluence in a 24 well plate were transfected with 50 ng of the pUC57-1.3mer-HBV D3 plasmid (accession numbers HBV X02496.1 and pUC57 Y14837.1) and 200 ng carrier pCMV3-C-FLAG plasmid (size matched to the HBV plasmid) using a Fugene 6 transfection protocol (Promega). After 24 h the transfection mixture was removed by washing with PBS and the cells cultured for 3 days, extracellular media collected and cells harvested for RNA extraction. For infection studies HBV genotype D3 was purified as previously reported [29]. Briefly, HBV was purified from the extracellular media of HepAD38 cells by heparin affinity chromatography and ultracentrifugation on a sucrose gradient. HBV stocks (>3×10^9^ viral genome equivalents [vge] ml^−1^) were stored at −80 °C. HepG2-NTCP cells were inoculated with HBV at a multiplicity of infection (m.o.i.) of 400 in the presence of 4 % PEG8000 and after 16 h the inocula removed by washing with PBS and cells cultured in the presence of 2.5 % DMSO. Media was replaced after 3 days and the cultures maintained for an additional 3 days, supernatants were collected and cells harvested for RNA extraction. Earlier studies showed maximal HBV RNA levels at 6 days post-infection in the *de novo* infection model and hence this time point was selected. In both transfection and infection experiments secreted HBe antigen was quantified by EIA (Autobio, PR China) on a BMG FluoStar Omega (BMG). Levels of HBeAg were interpolated from a standard curve, according to the manufacturer’s instructions.

### RNA purification, cDNA synthesis and qPCR

Cell lysates were prepared and RNA purified using an RNeasy kit in accordance with the manufacturer’s instructions (Qiagen). An RNase-free DNase was used for on-column removal of DNA from the HBV infected samples (Qiagen). The RNA from HBV1.3-transfected samples were further treated with TURBO DNase I (Invitrogen), according to the manufacturer’s protocol. RNA concentrations were measured using a Nanodrop 2000 spectrophotometer (Thermo Scientific). Up to 200 ng of RNA was reverse transcribed using a cDNA synthesis kit (PCR Biosystems) and HBV total RNA (F: ACGGGGCGCACCTCTTTA; R: GTGAAGCGAAGTGCACACGG) detected using a SYBR Green qPCR (SyGreen, PCR Biosystems) as previously described [30]. A β−2-Microglobulin gene was used as a housekeeping control (F: CTACACTGAATTCACCCCCACTG; R:ACCTCCATGATGCTGCTTACATG). HBV RNA copy numbers were calculated using a standard curve generated by serial dilution of a pHBV1.0 genotype D plasmid and all reactions included a no-reverse transcriptase control.

### Northern blotting for HBV RNA

Initially 10 µg of total RNA from HBV infected cells at 6 days post-infection were resolved on a 10 % MOPS, 2.2 M formaldehyde agarose gel. To assess RNA loading, the 18S and 28S ribosomal subunit RNA species were visualised under UV light through SYBR green staining. Gels were denatured in 50 mM NaOH for 5 min and RNAs transferred to nylon membrane by capillary transfer in SSC buffer. Membranes were washed and RNAs fixed by UV crosslinking. Membranes were hybridised at 65 °C overnight in the presence of a digoxigenin-labelled DNA probe of pHBV1.3, enabling the detection of all viral RNAs. Bands were visualised using a luminescent DIG detection kit (Roche) according to the manufacturer’s instructions. Images were collected with a PXi Touch Imaging system and band intensities quantified using built-in software (Syngene).

### HBV enrichment and PacBio sequencing

RNA quality was assessed using a Bioanalyzer 2100 (Agilent) and samples with an RNA integrity number higher than 8.5 selected for targeted amplification (Iso-Seq Express Template Preparation system, PacBio). One microgram of RNA was converted to full-length cDNA using a SMART cDNA synthesis kit (Takara Bio) and amplified using a HiFi PCR kit (Kapa Biosystems) using Iso-Seq Express amplification primers to incorporate PacBio sample indices or ‘barcodes’ (IDT). cDNA quantity and quality were determined using a Nanodrop 2000 spectrophotometer (Thermo Scientific) and a Bioanalyzer 2100 (Agilent). A panel of 120 nucleotide (nt) biotinylated oligos targeting the entire HBV genome were designed using the xGen Lockdown platform (IDT) and have been described elsewhere [31]. Briefly, probes were designed to match the consensus of 4499 HBV whole genomes in the Hepatitis B Virus Database (hbvdb.lyon.inserm.fr/HBVdb, genotypes: A 506, B 1218, C 1447, D 823, E 254, F 197, G 28 and H 26). We used RAxML to infer the ancestral sequences at the root of the tree and to design probe sequences with the lowest divergence from all possible isolates. Additional probes were synthesised for genotype G, which has a 36 base insertion in the core gene, and for genotype D that has a deletion of 33 bases in the pre-S1 region. Long capture probes of 120 nucleotides retain affinity for their target even with up to 20 % divergence [31, 32]. Probes were incubated with the pooled cDNA at 65 °C for 4 h. The biotinylated primer-cDNA complexes were incubated with Streptavidin Dynabeads at 65 °C for 45 min followed by washing and elution (xGen Hybridization kit, IDT). The captured DNA sequences were pooled and SMRTbell library ligation and amplification performed in accordance with the manufacturer’s instructions (PacBio). To determine the level of enrichment the pooled cDNA samples before and after oligo capture were amplified using a qPCR SyGreen mix (PCR Biosystems) with primers specific for total HBV RNA or β−2-Microglobulin as described above. Samples were sequenced using a Sequel II system (CCS protocol) to generate a PacBio ‘HiFi library’.

### Identification of HBV and human transcripts

PacBio SMRTLink software was used to demultiplex the HiFi reads. Sequences were aligned to HBV ayw genotype D (GenBank ID NC_003977.2 [10]), a linear 3581 bp genome (D3L) extending from the first TSS for preC (1525 [33]) to the TATAAA Poly Adenylation Site (PAS, 1918–1923), using the PacBio package PBMM2, a version of the minimap2 programme optimised for long read mapping (Supplementary Information: align-script.sh). This analysis was also performed using the human transcriptome as the alignment reference (Human GRCh38.p13 GenCode fasta file, www.gencodegenes.org) and sequences with a MAPQ score <30 discarded. CIGAR strings for each sequence were extracted from the BAM file and parsed using the GenomicAlignments package in R [34] (Supplementary Information: quantify_viral.R and quantify_human.R). Unspliced transcripts with starting coordinates mapping within 12 bases of the known TSS of the preC-L, preC-S, pgRNA, preS1, preS2, S or X and ending at the PAS were defined as canonical transcripts. Non-canonical or spliced transcripts were identified and intron junctions mapped to coordinates within 12 bases of known splice sites (GFF/GTF file from [33]). Splice patterns were checked by aligning to the HBV reference genome using Needleman Wunsch and Smith Waterman algorithms with either the R Biostrings package [35] or the EMBL Emboss server (https://www.ebi.ac.uk/Tools/emboss/). ‘Overlength’ transcripts were re-aligned to a 2 × overlength copy of the D3L reference sequence. Reads were normalised relative to the total number of sequences obtained (transcripts per million reads, TPM).

### Identification of HBV/human chimeric transcripts

Any flanking regions in the sequences that did not align to HBV (‘soft clipped regions’) were extracted and filtered, retaining only those >100 bp in length. These soft clipped regions were remapped to a fasta file containing the human genome (GRCh38.p13), the HBV D3L sequence and the pUC57 backbone of the HBV1.3 plasmid, using PBMM2. The Rsubread package was used to identify chimeric HBV/human transcripts (Supplementary Information: quantify_human_chimera.R).

### Differential analysis of viral transcripts

Differential analysis was performed using the R limma package Voom function to normalize the counts based on library size and transformed to log2 counts per million reads [36]. A linear model was fitted using weighted least squares for each gene, followed by empirical Bayes smoothing of standard errors [37]. Pairwise comparisons of viral RNAs between infected and transfected cells were performed.

### Data summary

All sequencing data are available to be downloaded at NCBI (BioProject PRJNA929325, accession numbers SAMN32957167, SAMN32957168, SAMN32957169 and SAMN32957170). All code and analysis scripts are open source and can be found in the supplementary methods.

## Results

### Probe-based sequence capture and long read sequencing of the HBV transcriptome

**Fig. 1.**
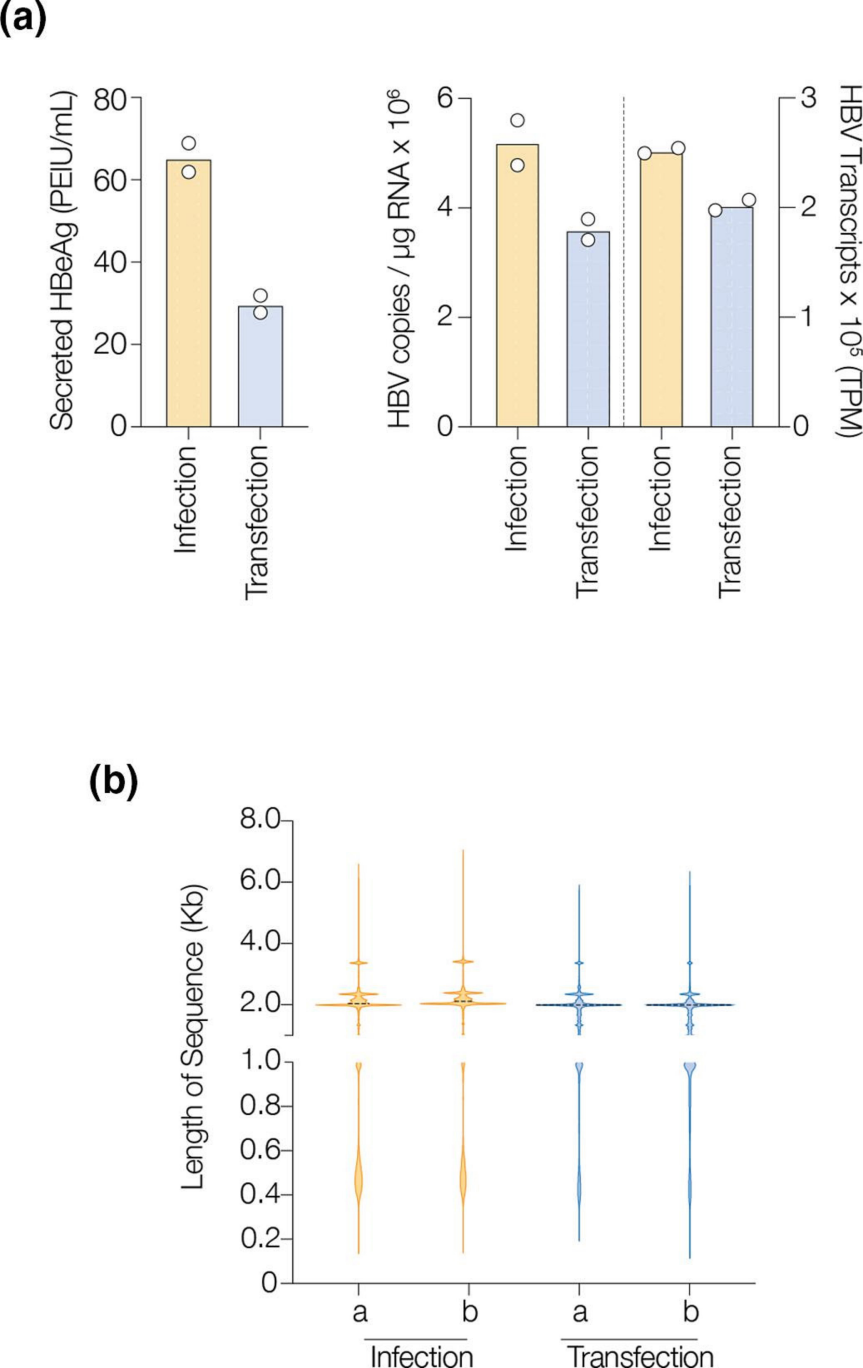
Preparation and enrichment of HBV cDNA for sequencing. (**a**) Secreted HBeAg in HBV infected (m.o.i. 400) or HBV1.3 transfected HepG2-NTCP cells (HBV1.3) (left). RT-qPCR quantification of HBV RNAs or number of viral transcripts per million reads (TPM) detected by sequencing of infected or transfected samples (right). (**b**) Violin plot of HBV RNA lengths in infected and transfected HepG2-NTCP cells. Peak sizes corresponding to 3.5 Kb (preC/pg RNA), 2.4 Kb (preS1) and 2.1 Kb (preS2) are clearly visible.

To generate the templates for PacBio sequencing we infected or transfected HepG2-NTCP cells with HBV genotype D (strain ayw) and, after 6 or 4 days respectively, measured levels of secreted HBeAg and total viral RNA. Optimising the amount of HBV plasmid used to transfect cells resulted in similar levels of viral RNA and antigen expression to the *de novo* infected samples (Fig. 1a). qPCR measurement of HBV RNAs showed 3–5×10^6^ copies per microgram total cellular RNA, demonstrating that <1 % of cellular RNA is of viral origin. These low levels of HBV RNA are consistent with earlier studies analysing *in vitro* model systems and clinical samples [10, 15, 38, 39]. To overcome the challenge of low RNA levels in HBV infected material for long-read sequencing we used a probe-based capture method to enrich the viral RNAs. RNA was reverse transcribed to cDNA, amplified using Iso-Seq Express primers to incorporate multiplex PacBio ‘barcodes’ and checked for quantity and quality before pooling and oligo-nucleotide enrichment with the xGen Lockdown platform (IDT). qPCR enumeration of HBV RNA in the pooled cDNA library before and after oligo-probe enrichment showed an approximate 700-fold enrichment of viral transcripts. These data show that oligo-probe capture can increase the abundance of templates for long-read sequencing where viral transcripts may be limiting. To assess whether the binding of RNA to the capture beads could lead to strand breakage we identified the start coordinates of all transcripts and found that >98 % of RNAs mapped to within 50 nucleotides of a known TSS, suggesting low levels of strand breakage. HBV enriched samples were sequenced following a standard PacBio IsoSeq protocol in a SMRT Cell 8M cartridge. Following demultiplexing of the barcodes, the reads were mapped against the HBV genotype D3L reference sequence using the PBMM2 software (Supplementary Information: align-script.sh). Aligning the reads in the HBV infected and transfected samples identified an average of 25 and 20 % of sequences as viral, respectively. To ensure correct assignment of reads, we selected sequences that contained whole transcripts, starting at a known TSS and terminating at the PAS site (TATAAA). We found very few transcripts (17 among all four sequenced sample sets) terminating at the reported cryptic PAS (CATAA at 3448–3453). The frequency of HBV sequences were expressed as transcripts per million reads (TPM) and reassuringly these counts are consistent with our earlier RT-qPCR quantification and with northern blot analysis (Figs 1a and S1, available in the online version of this article). The distribution of transcript lengths in samples from both model systems were comparable, with peaks at 3.5 Kb (preC and pg), 2.4 Kb (preS1) and 2 Kb (preS2, S) (Fig. 1b). Although some shorter transcripts are apparent, they comprise less than 4 % of the viral RNAs and there was no distinct 0.7 Kb peak that would correspond to the HBx transcript. As the protocol for preparing templates for PacBio sequencing includes two column purification steps, designed to remove excess primers and short transcripts (< 1 Kb), this will likely influence our results. However, our data are consistent with earlier studies that showed a low abundance of HBx RNAs by Illumina short-read sequencing or 5′RACE methods [8, 10, 40].

### Mapping HBV transcripts in infection and transfection model systems

**Fig. 2.**
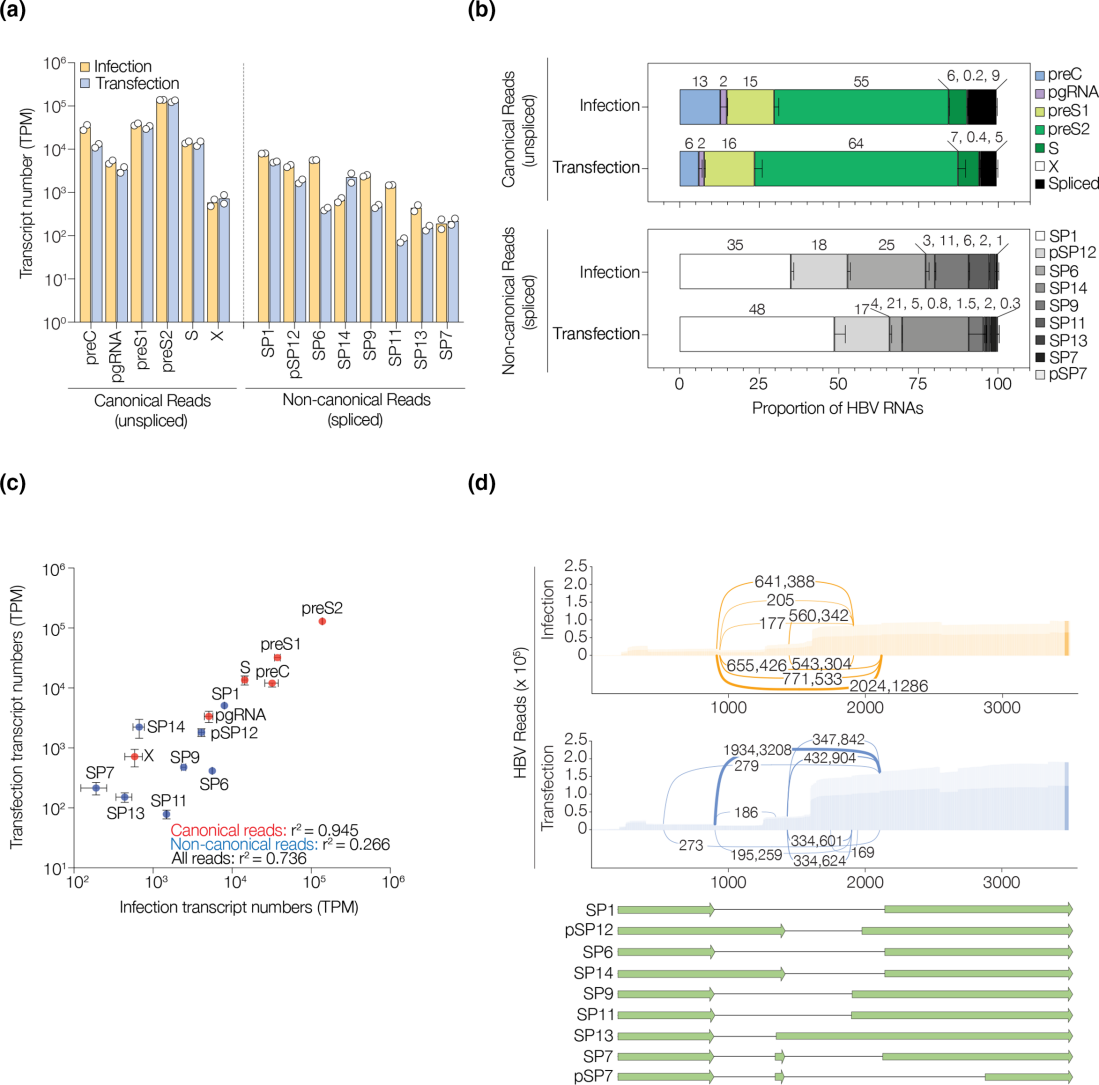
PacBio analysis of HBV transcriptomes. (**a**) Frequency of canonical and non-canonical (spliced) HBV transcripts in infected or transfected HepG2-NTCP cells presented as transcripts per million (TPM), where the data is from two biological replicates. (**b**) Relative frequency plots of individual canonical and non-canonical HBV RNAs isolate from infected or transfected cells. (**c**) Correlation of canonical (red) and non-canonical (blue) RNAs in the two models. (**d**) Sashimi plots denoting the location of splice donor and acceptor sites and number of spliced transcripts in infected or transfected HepG2-NTCP cells, with a cartoon of the spliced transcripts identified in these samples.

The location and splicing patterns of the HBV reads were extracted (Supplementary Information: quantify_viral.R) and classified according to their transcription start and end coordinates, and the presence or absence of splicing. HBV transcripts were classified as canonical (unspliced) or non-canonical (spliced) using previously reported nomenclature (Table 1, Fig. 2a) [10, 33]. The limma software package [41] was used to compare viral transcripts between the infection and transfection samples. Comparable patterns of pg, preS1, preS2, S and X RNAs were detected in both models, with preS2 being the most common viral RNA (Fig. 2a, b). We noted significantly more preC in infected (13%) compared with transfected (6 %) cells (adjusted p value<0.05). The longer form of the pre-C transcript (preC-L, starting at 1525 in the HBV genome) was relatively rare compared to the shorter (pre-C-S) form (Table 1). Overall, the infected and transfected cells showed a similar pattern of canonical transcripts (r^2^=0.945, Fig. 2c), whereas greater variation was observed in the non-canonical transcripts (r^2^=0.266, Fig. 2c). Among the previously described non-canonical transcripts SP1 was the most abundant, representing up to 48 % of the spliced RNAs. We detected seven spliced RNAs that constituted greater than 0.1 % of the viral transcriptome, of which SP6, SP9 and SP11 were more frequent in the infected samples, whereas SP14 was more abundant in the transfection model (adjusted p values<0.05, Table 2, Fig. 2a, b, Supplementary Information: differential_virus.R). In support of these data, sashimi plots show a similar usage of splice donor and acceptor sites between all samples (Fig. 2d). Collectively these results show comparable viral transcriptomes in these two infection models.

**Table 1.** Number of HBV transcripts detected in each sample

	Sample ID	Infection-a	Infection-b	Transfection-a	Transfection- b
	Barcode	1002	1003	1010	1012
Canonical Transcript counts:					
	preC-L*	359	257	471	678
	preC-S	10 541	5262	6048	7535
	pgRNA	1339	1050	1765	1978
	preS1	10 184	7554	15 805	20 375
	preS2	40 140	26 097	55 901	94 134
	S	3954	2878	5426	10 542
	X	139	131	251	608
Total canonical reads :		66 656	43 229	85 667	135 850
Non-canonical Transcript counts :					
	SP1	2323	1508	2251	3618
	pSP12	1253	729	743	1393
	SP6	1608	1072	175	309
	SP14	215	112	764	1920
	SP9	736	443	236	302
	SP11	432	278	40	48
	SP13	147	70	60	118
	SP7	69	27	114	124
	pSP7	0	0	25	1
	SP18	0	5	0	1
	90009_ccs	0	0	8	0
	27415_ccs	0	3	0	0
	SP5	0	0	2	0
	7169_ccs	0	0	0	4
	107477_ccs	0	1	0	0
Total non-canonical reads:		6783	4248	4418	7838
As % of total HBV reads:		9.24	8.95	4.90	5.45
Total # of HBV reads:		73 439	47 477	90 085	143 688
Total # of all sequences:		288 678	190 141	456 085	693 503
Percentage HBV reads:		25.4	25.0	19.8	20.7

**Table 2.** Differential expression analysis of HBV transcripts for infection vs transfection

Transcript	logFC	AveExpr	t-test	P.value	adj.P.val
SP6	3.277	12.728	8.855	<0.001	<0.001
SP11	3.803	10.573	6.452	<0.001	<0.001
SP9	1.960	12.198	5.067	<0.001	<0.001
SP14	−2.172	12.376	−5.021	<0.001	<0.001
preC-S	0.938	16.406	2.762	0.012	0.044
pSP12	0.702	13.563	2.045	0.053	0.145
SP13	1.044	10.151	1.774	0.090	0.214
X	−0.759	11.462	−1.701	0.103	0.218
preS2	−0.375	19.171	−1.269	0.218	0.415
S	−0.364	15.925	−1.152	0.262	0.435
SP7	−0.596	9.799	−0.898	0.379	0.529
preS1	−0.236	17.216	−0.767	0.451	0.536
SP1	0.187	14.792	0.582	0.567	0.633
preC-L	−0.183	12.427	−0.512	0.614	0.648
pgRNA	0.136	14.145	0.413	0.684	0.684

### Quantification of overlength and chimeric transcripts

**Fig. 3.**
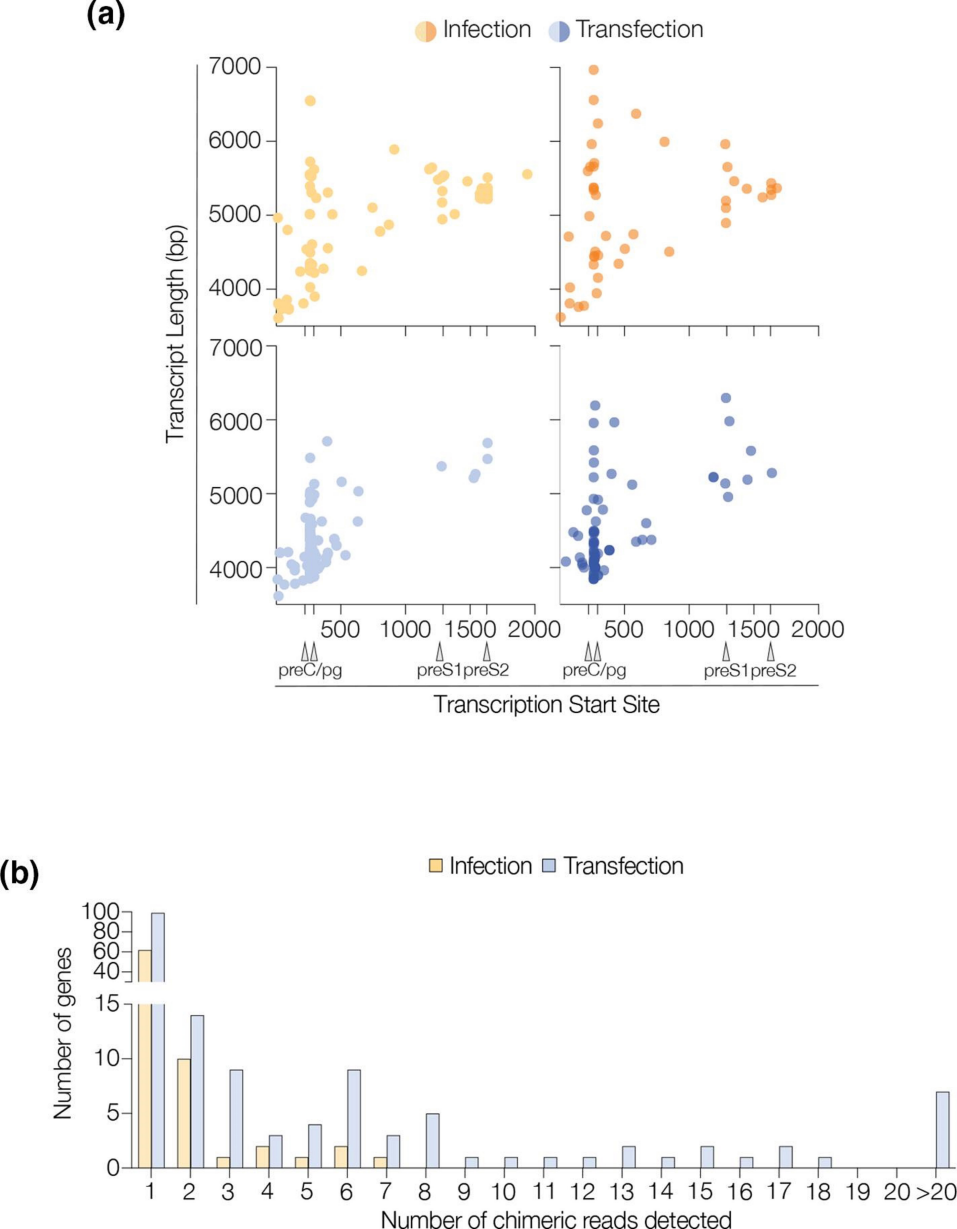
Detection of overlength or viral-chimeric transcripts. (**a**) Start coordinates and lengths of transcripts which do not terminate at the PAS (‘overlength’ transcripts, replicates shown separately). Approximately ~1 % of transcripts are of this type, with the majority starting at either the first or second transcription start site for preC/pg RNA. TSS are annotated. (**b**) Number of HBV-human chimeric RNAs quantified in the infected or transfected cells, the complete list of all human gene chimaeras detected are in Supplementary Table 1.

A minor fraction (~1 %) of ‘overlength’ viral transcripts were detected in both model systems, where the 3′ end of the RNA occurred after the end of the HBV D3L reference sequence. None of the transcripts have the predicted dslDNA terminus, suggesting they are not derived from chromosomal integrants. The majority of these transcripts started at the two major TSS for preC and pg RNA (positions 223 and 292) and extended beyond the PAS (Fig. 3a). We also identified chimeric sequences, comprising transcripts that mapped to both the viral and human reference genomes. Sequences that aligned to HBV, but that included >100 bp regions of non-viral origin were investigated. These non-viral regions (Supplementary Information: extract_soft_clipped.R) were aligned to the human transcriptome (Supplementary Information: remap_soft_clipped.sh) and quantified (Supplementary Information: quantify_human_chimera.R). We observed fewer chimeric reads in the infected cells (TPM: 259 and 220) compared to the transfected cells (TPM: 442 and 776), however, this difference was not significant (*P*=0.27) (Fig. 3b). In the HBV transfected samples several thousand chimeric reads contained regions that mapped to the plasmid vector (HBV1.3a, 6976 reads, HBV1.3b 7975 reads) indicating transcription directly from the plasmid.

**Fig. 4.**
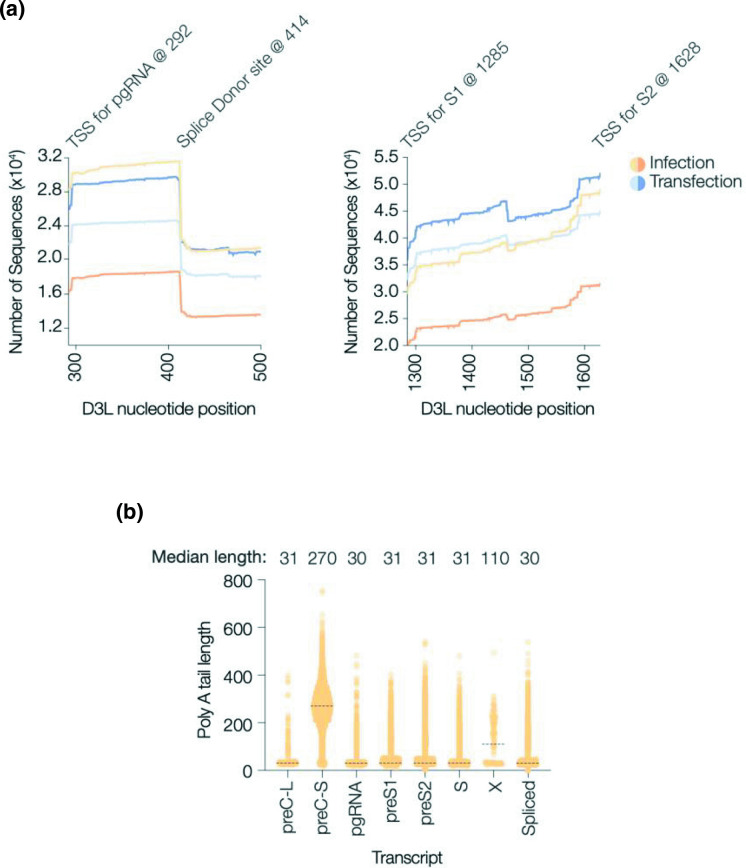
Properties of HBV Transcripts. (**a**) Coverage plot (no. sequences aligned to each base of the D3L reference genome) for the region downstream of the TSS for pgRNA (left panel) and for preS1 (right). The TSS are annotated along with the major splice donor site. The preS1 region includes the splice donor site for SP14 and pSP12 at position 1463 along with the acceptor and donor sites for the second exon of SP7 at positions 1380 and 1463 respectively (coordinates 2935 and 3018 of [10]). (**b**) PolyA tail lengths of individual non-spliced transcripts are presented and data pooled for the spliced transcripts. For each transcript the median length of the polyA tail is shown. The data shown is for the ‘Infection-a’ sample, the other three datasets gave similar values.

In this data set several thousand reads mapped to every position of the D3L reference sequence. Inspecting the sorted alignments using Ugene software [42] identified truncated forms of some transcripts, consistent with 5′−3′ degradation events. In all samples we noted an increasing number of transcripts mapping toward the 3′ end of the region between the TSS for preS1 and preS2, with 30 % more reads mapping immediately upstream of the TSS for preS2. However this gradient was not observed for pgRNA, possibly reflecting the stabilisation of this region by the 5′ epsilon structure (Fig. 4a). This observation is consistent with exosome mediated degradation of HBV RNAs [43]. Mapping the polyadenylation length (Supplementary Information: extract-seq.sh and Acounter.R) showed a median of 29–31 for all HBV RNAs with the exception of preC-S, where the median length was >200 nucleotides, reflecting a population of short transcripts that terminate at the PAS (Fig. 4b). As polyadenylation is known to regulate RNA half-life and translation, these intriguing results are worthy of further investigation.

## Discussion

We have developed a protocol for the enrichment of HBV RNAs obtained from experimental infections and a sequence analysis pipeline to quantify the viral transcriptome. The use of a high fidelity long read method solves one of the more intractable problems inherent in more conventional short read approaches, that of sequencing full length transcripts. Although short read sequencing allows the construction of whole genome consensus sequences and quantification of intrasample diversity and polymorphism in short regions of the genome, it cannot capture entire HBV transcripts and offers a limited insight into the relative activity of the major viral promotors. The short length of sequences obtained from Illumina sequencing (typically ≤300 bp) can also be problematic when exploring the patterns of HBV RNA splicing. Although individual splicing events are readily detectable, the assignment of multiple splicing events is unreliable [10]. Long read sequencing approaches have been available for several years [21, 22, 44], however it is only recently that their accuracy has improved to a level (>99 %) where they offer an alternative to short read methods. The PacBio sequencing platform is becoming more widely available and, combined with approaches that allow for multiplexing of samples, is financially competitive with more established platforms.

We developed a panel of 74 biotinylated DNA oligonucleotides, where each 120 nt sequence overlaps the next by 60 nt and target diverse viral genotypes, that enriched HBV cDNA in the NGS libraries by approximately 700-fold. Without this enrichment step, we would have to generate several hundred fold more sequences to obtain the same number of HBV transcripts. Our analysis pipeline could be applied to the conceptually similar Nanopore rolling circle sequencing method and modified to study the transcriptome of other viruses. Although the number of sequences obtained by the Nanopore method is lower than the PacBio platform (500–2600 total sequences, depending on sequencer flow cell), if 20–25 % of post-enrichment reads are of viral origin, then 100–500 full length HBV reads could be obtained.

We showed a similar pattern of canonical transcripts in both model systems, however we noted differences in the relative abundance of spliced RNAs. SP1 was the most abundant spliced transcript in both model systems, consistent with earlier reports [10, 27, 45]. The pattern of spliced RNAs in the transfected cells are comparable to previously published work from Lim and colleagues [10]. Long-read sequencing methods allowed us to make several interesting observations. Firstly, we were able to determine the frequency of overlength reads and map their start and end points (Fig. 3a). Secondly, we were able to exploit the great depth of coverage to infer the occurrence of 5′−3′ transcript degradation and to suggest that RNAs may differ in their susceptibility to degradation (Fig. 4). Finally, the ability to sequence full-length RNAs allowed us to measure their poly A tails, with the surprising observation that preC-S had a significantly longer polyA tail. Although longer than the other viral transcripts (>200 vs 30), the observed polyA tail of preC-S is typical of many eukaryotic transcripts [46, 47].

We found 75–80 % of the RNAs in this dataset mapped to the human genome, however as the NGS library was generated by HBV specific capture enrichment this could influence the relative abundance of host transcripts. The residual host transcripts may have some degree of homology to the capture probes, be of the right size, GC content, or have some other property that allows them to persist through the enrichment process. Given these limitations we could not reliably investigate the human transcriptome in these samples.

In summary, capture-based enrichment combined with accurate long read sequencing offers the potential to obtain the sequence of individual transcripts, complex splice variants and viral-human chimeric mRNAs, allowing the accurate quantification and mapping of the HBV transcriptome. This method offers an advance over short read approaches and will facilitate in depth analysis of HBV transcription in liver biopsy samples.

## Supplementary Data

Supplementary material 1
